# The Quality of Life of People with Solid Cancer is Less Worse than Other Diseases with better Prognosis, Except in the Presence of Depression

**DOI:** 10.2174/1745017902117010315

**Published:** 2021-12-31

**Authors:** Cesar Ivan Aviles Gonzalez, Matthias Angermeyer, Laura Deiana, Caterina Loi, Elisabetta Murgia, Anita Holzinger, Giulia Cossu, Elena Massa, Ferdinando Romano, Mario Scartozzi, Mauro Giovanni Carta

**Affiliations:** 1 Universidad Popular del Cesar, Valledupar, Colombia; 2 Center for Public Mental Health, Gösinhg am Wagram, Austria; 3 Department of Medical Sciences and Public Health, University of Cagliari, Cagliari, Italy; 4 Medical University of Vienna, Vienna, Austria; 5 Department of Public Health and Infectious Diseases, University of Rome “La Sapienza”, Rome, Italy

**Keywords:** Health-related quality of life, Oncology, Psychosocial health, Depression, Psychiatric comorbidity, Psycho-oncology

## Abstract

**Background:**

Suffering from Solid Cancer (SC) may adversely impact the Health-related Quality of Life (H-QoL). The aims of this study are to measure the H-QoL in a sample of people suffering from SC and to clarify the role of the co-occurrence of depressive episodes. Results were compared with a healthy control group and with groups of other disorders.

**Methods:**

In 151 patients with SC (mean±sd age 63.1±11.5; female 54.3%), H-QoL was assessed by SF-12, depressive episodes were identified by PHQ-9. The attributable burden of SC in impairing H-QoL was calculated as the difference between SF-12 score of a community sex and age ¼ matched healthy control group and that of the study sample. The attributable burden of SC was compared with other chronic diseases using specific diagnostic groups drawn from case-control studies that used the same database for selecting control samples.

**Results:**

H-QoL in people with SC was significantly worse than in the healthy control group (p<0.0001). The attributable burden in worsening the H-QoL due to SC was similar to those of severe chronic diseases, but lower than Multiple Sclerosis (p<0.0001) or Fibromyalgia (p<0.00001). Having a depressive episode was a strong determinant of decreasing H-QoL, regardless of the severity of cancer.

**Conclusion:**

The findings confirm a strong impact of SC but showed that H-QoL in SC was higher than in chronic diseases with better “quoad vitam” outcome. Since depression was a strong determinant, its prevention, early detection and therapy are the main objectives that must be reached in cancer patients.

## 1 INTRODUCTION

According to the World Health Organization, 9.6 million people worldwide died from cancer in 2018 [1]. Considering the progressive ageing of the population worldwide [2] and that most forms of cancer have the highest frequency among the elderly, cancer remains a major public health issue [3].

Receiving the diagnosis of cancer, as well as being in long-term and painful treatments, adversely impact the Health-related Quality of Life (H-QoL) and the psychological wellbeing of people suffering from cancer [4-12].

H-QoL is a complex and multidimensional construct that doesn’t simply deal with the concept of standard of living, which primarily concern income and employment status [13]. In fact, indicators of subjective perception of H-QoL range between the built environment, physical and mental health, education, including elaborate psychological components of wellbeing like, satisfaction with recreation and leisure time, social belonging satisfaction with cares, mood, sleep and rest, energy, vitality [14, 15].

Research evidence show that H-QoL may predict survival in people with several kinds of cancer [16] and could be a prognostic factor of clinical outcome and adherence to treatment [14, 16-18]. Thus, the measure of H-QoL in people suffering from chronic diseases such as solid cancer has become relevant in both prognostic and outcome indicators flanking the traditional outcomes measures, such as adherence to treatments, therapy response and survival rates, to compare different treatment strategies in clinical trials and cohort studies [10, 11, 16, 18-20]. Hence, interest in factors associated with enhancing H-QoL, alleviating psychological distress and facilitating positive adaption to the illness has increased [21, 22], as well the search for accessible and simple ways to measure the H-QoL of large patient cohorts such as the adoption of e-Health based instruments [23, 24].

Among factors associated with a lowering of the H-QoL, an important role has been recognized in several chronic diseases at the co-occurrence of Major Depressive Disorders [25-29]. Although some research has sought to elucidate the relationship between Major Depressive Disorders, H-QoL and cancer [18, 30, 31], this relationship has so far been little studied.

The primary outcome of the present study is to assess H-QoL in patients diagnosed with solid cancers and compare the results with the general population and with other chronic pathologies. Furthermore, another aim is to clarify the role of depressive episodes and the relationship between cancer stage and depression and H-QoL.

## 2 METHODS

### 2.1 Design

Cross-sectional study, with a case-control analyses on selected sub-samples.

### 2.2 Recruitment and Study Sample

The study sample included patients consecutively recruited during June-July 2018 at the Oncology Unit of the University Hospital of Cagliari, Italy.

The inclusion criteria was: ≥18 years old ageing; both genders; with a histologic confirmed diagnosis of malignant neoplasia in treatment; signature of the written informed consent form.

### 2.3 Tools and Assessment

 The Italian version [32] of the self-report questionnaire SF-12 (Short Form Health Survey – 12 item) [33] was used to evaluate the H-QoL. The instrument consisting of 12 items and investigate two sub-dimensions: physical and psychosocial health. The total score (range: 12-47) resulting from answers given by several Likert scales. Higher scores reflect a better perceived H-QoL.

 The Italian Version [34] of the Patient Health Questionnaire [35] was used to identify Depressive Episodes. The PHQ-9 is a self or phone-administered easy to use screening questionnaire. It detects each of 9 DSM core criteria for a major depressive episode in the form of specific questions; the scores range for each item from “0” (complete absence) to “3” (almost every day), with “1” (a few days and) and “2“ (more than half of days). The score resulting from the sum of the value of each item is classified: from 0 to 4 for as minimal symptoms; 5-8 subthreshold depression; 9-14, mild depression; 15–19 moderately severe and from 20 to 27 severe [36].

The score of 10 is the optimal cut-off for identifying depressions of clinical relevance in two-phase investigations; the cut-off 14 is more reliable in case finding investigations because the greater reliability of “positive” as “cases” (decreasing in false positives) allows better study of the associated factors [37].

*Ad-hoc* form to collect socio-demographic and clinical-oncological anamnestic data, such as: gender (M/F); age; marital status (single, married, divorced, widowed); employment status (part-time, full-time, unemployed, student, housewife, retired, other); educational level (primary, secondary school, high school, university, higher eg, Master's degree or PhD); kind of the oncology service (Day Hospital – out-patients/Hospital Ward – in-patients; time from taking in charge of the patient at the Oncology Unit (first visit, <6 months, 6-12 months, >12 months); cancer site (gynecological, urogenital, gastroenteric, rare cancers); cancer stage (from I to IV, progressively indicating a worse clinical status of the patient); intent of treatment (adjuvant, neo-adjuvant, palliative), toxicity of treatments (scored from 0 (mild) to 5 (death)), according to Common Toxicities Criteria for Adverse Events (CTCAE) [38], adherence to treatment (yes/no), response to treatment by computerized axial tomography (CAT) according to RECIST criteria (Complete Response -CR, Partial Response-PR, Stable Disease -SD, Progression of Disease -PD). The term “Ongoing” was used to describe patients who had not been assessed for the response to treatment yet (for example, because they have not yet completed the expected number of cycles) [39].

### 2.4 Ethical Aspects

The study was approved on September 26, 2018, by the Ethical Committee of the Sardinia Region, Italy and registered with the number PG/2018/13269. All procedures were carried out in accordance with the 1964 Helsinki Declaration and its later amendments. All the patients of the study sample signed a written informed consent after a full description of the aims, the procedures, the data protection and the possibility to terminate the study at any time.

### 2.5 Statistical Analyses

Data were anonymously collected by an ID number for each subject and data-entry was made in a dedicated database. Data were analyzed with the Statistical Package for Social Science (SPSS) for Windows (Chicago, Illinois60606, USA), version 21.

The descriptive statistics (mean±sd; frequency N and percentage %) were used for continuous and nominal variables, respectively, to point out the socio-demographic and clinical-oncological anamnestic characteristics of the samples.

The one-way ANOVA statistic was used to verify the association between variables regarding socio-demographic and clinical-oncological anamnestic data and H-QoL in the Study Sample (SS), as well as the association between H-QoL and depressive episodes, controlling for the role of cancer stage in this association.

The attributable burden of SC in impairing H-QoL was calculated as the difference between SF-12 score of a community sex and age ¼ matched healthy control group sample and that of the study sample. Being diagnosed with cancer with all that derives puts patients at risk for depression; depression affects a proportion of 15-25% of patients with cancer and 4.4%, of the general population [8, 9]. Having cancer and being exposed to depression is an associated worsening quality of life condition. For the aim of creating the control group, a block was created for each subject with cancer and four individuals per block of all eligible ages (±2 years) and of the same gender were extracted from the database of a community survey on well-being in the Italian population [40]. Once drawn out end was included in a block, they were automatically excluded from the remaining blocks.

The attributable burden of SC was compared with other chronic diseases using specific diagnostic groups drown from case-control studies that used the same database for selecting control samples [26-28, 41-46], as well as for the choice of the Healthy Control Sample (HCS).

The H-QoL as SF-12 scores in groups with or without Major Depression, as well as in a different stage of cancer in the Study Sample (SS), were compared using analysis of variance (one-way ANOVA).

Finally, we calculated the burden attributable specifically to Depression as the mean difference in the SF-12 score of people with Solid Cancer without depression from the mean SF-12 score of people with Solid Cancer and depression. A similar procedure had also been carried out in the aforementioned case-control studies that had used the same database for the construction of the control groups; it was, therefore, possible to compare the burden attributable to having Major Depressive Disorder in Cancer with that which occurs when people have Major Depressive Disorder in other chronic diseases.

## 3 RESULTS

The SS consists of 151 people, including 82 females (54.3%) and 69 males (45.7%). The matched CS from a sample of people declaring they do not have cancer and are representative of the Italian population [39] include, 604 people, 328 females (54,3%) and 276 males (45.7%). The mean±sd age of the SS was 63.14±11.548 years and 63,31±11.718 years for the CS (Table **1**). The socio-demographic and clinical-oncological characteristics of the SS are illustrated in Table **2**. In the SS, the mean±sd of the SF-12 total score was 32.34±6.764; in the CS the mean±sd of the SF-12 total score was 37.01±6.561, the difference reached the statistically significant difference of (F= 60.539, df 1,753,754, p <0.000).

As shown in Table **3**, the impact on H-QoL of the burden attributable to the solid cancer was 4.67±6.64 points (as the difference between healthy control matched sample and Study Sample on the SF-12). The H-QoL profile in patients diagnosed with a solid cancer was comparable to that with other chronic diseases such as Major Depression (F=0.6753; df=1,186,187; p=0.4123), Wilson’ Disease (F=0.0375; df=1,172,173; p=0.8467), Carotidal Atherosclerosis (F=2.0799; df=1,195,196; p=0.1509), Obsessive Compulsive Disorder (F= 1.619; df 1,175,176; p=0.205), and Post-Traumatic Stress Disorder (F= 0.347; df 1,175,176; p=0.557). The impact on H-QoL was significantly greater than that due to Celiac disease (F=6.9304; df=1,209,210; p=0.0091) and to Specific Phobia (F=10.497 df 1,77,178

p=0.001) and significantly lower than that due to Multiple Sclerosis (F=18.0766; df= 1,350,351; p=0.000) and Fibromyalgia (F=48.8075; df= 1,220,221; p=0.0000).

Table **4** shows the Association between H-QoL (as a score of SF-12) and moderate-severe depressive symptoms (PHQ-9≥14). The table also shows the difference in the staging of tumors (mean±standard deviation, stage 1, 2, 3, 4) in the two groups of people with and without depression, to verify the possible confounding factor due to a different level of severity in the two groups (*i.e.*, that the different level of depression and consequently the different quality of life may depend on the different severity of the tumors). The two samples are perfectly balanced as regards the distribution of stage severity of the tumors (F=0.59; p= .690, mean differences 0.07).

Table **5** shows the Attributable Burden on worsening H-QoL due to Major Depressive Disorder in people with solid cancers in comparison to the Attributable Burden on worsening H-QoL due to Major Depressive Disorder in other chronic diseases. Attributable burden caused by Major Depressive Disorder in people with solid cancer is higher than the burden due if having Major Depressive Disorder in all chronic diseases examined: Multiple Sclerosis (F = 98.85, 1,350,351 df, p <0.0001); Fibromyalgia (F = 47.27, 1,220,221 df, p <0.0001); Wilson Disease (F = 19.56, 1,150,156 df, p <0.0001); Celiac Disease (F = 61.09, 1,201,210 df, p <0.0001); Carotid Atherosclerosis (F = 39.07, 1,195,196 df, p <0.0001).

## 4 DISCUSSION

The present study shows that people with solid cancers have significantly worse H-QoL than people living in the community without cancer, confirming some previous pieces of evidence [4, 47-49]. The result partially disagrees with the work of Lee and Cartmell [50] in which scores on PHQ-9 (the same tool used by our study to measure depression) and EQ-5D (a H-QoL measure) reported by cancer survivors which were not different from the general population.

As already pointed out in a recent study regarding hematological cancers [4], another important finding of the present research is that solid cancers have a similar impact on the patient H-QoL, along with other chronic diseases such as Major Depression, Wilson’ Disease, Carotid Atherosclerosis, Obsessive Compulsive Disorder and Post-Traumatic Stress Disorder. A significantly higher impact of solid cancers on H-QoL was reported compared with Celiac Disease and Specific Phobia, but lower compared with Multiple Sclerosis and Fibromyalgia [26-28, 41-46]. Despite the considerable impact that having a tumor can imply on the life of an individual, nonetheless highly disabling chronic diseases that do not have an equally “quoad vitam” prognosis, such as Multiple Sclerosis [51] or Fibromyalgia [52], present a burden attributable to worsening the H-QoL much higher than solid cancer.

In this respect, it is interesting to note that Fibromyalgia, as cancers and their treatments, is connoted by high levels of perceived pain [53], such that the significantly higher level of the impact on H-QoL observed by the comparison with that of solid cancer could be due to the different nature of somatic, cognitive and emotional components of pain in Fibromyalgia [54] than those in cancer [55, 56].

The fact that fibromyalgia only recently in the biomedical field and in the regulations concerning the recognition of the condition of disability caused by somatic diseases has been identified as “true” illness may have caused that situation of “being strongly misunderstood”, which has been complained of by people with Fibromyalgia [52, 57]. On the other hand, being affected by a tumor is probably less stigmatizing, unlike what happened a few decades ago, also in relation to the improvement of treatments and the possibility of a good outcome. In fact, more and more often we read of show media and business people, politicians or, in any case, people in view of public opinion who speak openly about their events related to a cancer diagnosis, while until a few years ago, all this was hidden. This may have improved the impact of the disorder [58].

The presence of a depressive episode lowers the SF-12 score on average by 10 points in people with solid cancer regardless of the level of cancer staging; thus, the study indicates that comorbidity with a depressive episode is a powerful factor associated with worsening H-QoL in people with solid tumors. This potential of the depressive episode to worsening the H-QoL is much higher in tumors than in any other chronic disease examined.

We hypothesize that living with solid cancers poses the problem of the awareness of the possibility of the end of life, and in this condition, the equilibrium can be governed on the one hand by a renewed hope in survival due to the improvement of treatments and on the other by the acceptance of the end of life when the health worsens, the onset of a depressive episode can catastrophically break this balance more than in other disorders where the worsening is given by pain, functional disability and stigma (factors having a role even in cancer but in addition of the above described condition). However, future studies will have to clarify these aspects, verify the hypothesis and possibly generate others. To our knowledge, ours is in fact, the first study that highlights these very important aspects.

The causal direction of the association between depression and low H-QoL is not obvious. If it is intuitive, but also reported by the literature, that a depressive episode can dramatically lower the H-QoL of an individual [59], it is also plausible that the stressful impact of having a tumor may be more important in a person who perceives a low H-QoL [60]. It is, therefore possible that a person who receives the diagnosis of cancer and lives the daily life of the struggle for survival and seeks to cope in the new condition may be more vulnerable to depression if, even before the tumor event, he perceives a low H-QoL.

However, while it is very difficult to identify effective tools to modify the perception of H-QoL, the depressive episode can be prevented and cured, and it is shown that the improvement of depression leads to an improvement in the perception of H-QoL [61].

Therefore, considering that both factors (depression and low H-QoL) have been associated with a worsening of the outcome. Prevention, early identification and therapy of the depressive episode become essential in people with solid cancer.

The study also suggests that in people with solid tumors, it is essential to identify the conditions of compromise in H-QoL and to take charge of these aspects, firstly by verifying whether depressive symptoms coexist.

The present study has several limitations. The first one regards the small sample size that decreases the power of the difference in the associations between H-QoL and Depressive Episodes. Another limitation is relative to the observational nature of the cross-sectional study that does not allow to evaluate the causal direction of the association links (*i.e.*, between depression and low H-QoL). Cohort studies on large samples are needed to consolidate the evidence and clarify if the SF-12 score is comparable among cases and controls but apparently not among pathologies without taking into consideration some potential risk factors that characterize each pathology, such as the different gender and age distribution or different comorbid conditions. However, having conducted the comparison not between different pathologies directly, but between differences for each specific pathology of the SF-12 in a sample with diagnosis and with a specific control group extracted from the same database but balanced for sex and age on the specific pathology, may have attenuated this potential bias.

## CONCLUSION

The study confirms a strong impact of solid cancer, but also shows that H-QoL in solid cancer was higher than in chronic diseases with better “quoad vitam” outcome. Since depression was a strong determinant, its prevention, early detection and therapy are the main objectives that must be reached in patients with cancer.

## Figures and Tables

**Table 1:** Matching by gender and age between study sample (SS) and a healthy community sample (HCS) from the Italian general population*.

**STUDY SAMPLE**				CONTROLS from community sample of the Italian general population*			
**Gender**							
	**N**		**%**	-	**N**		**%**
**Female**	82		54.3	**Female**	328		54.3
**Male**	69		45.7	**Male**	276		45.7
**Total**	151	100.0		**Total**	604		100.0
**Age**							
**N**	**mean**	**sd**		**N**	**mean**	**sd**	
151	63.14	11.548		604	63.31	11.718	

**Legend. **N= frequency; %= percentage; sd= standard deviation

*[Carta *et al.*, 2010]

**Table 2:** Socio-demographic and clinical characteristics of the study sample.

**VARIABLE**		**N**	**%**
**Gender**	Male	69	45.7
	Female	82	54.3
**Marital status**	Single	31	20.5
	Married	102	67.5
	Divorced	5	3.3
	Widow	13	8.6
**Employment status**	Housewife	32	21.2
	Unemployed	13	8.6
	Employed	49	31.5
	Retired	57	37.7
**Education level**	Primary school	24	15.8
	Secondary school	54	35.8
	High school	54	35.8
	University degree	17	11.3
	Higher	2	1.3
**Kind of service**	Day Hospital	118	78.2
	Hospital Ward	33	21.9
**Timing of taking care at the Oncology service**	First visit	7	4.6
	<6 months	71	47.0
	6-12 months	29	19.3
	>12 months	44	29.1
**Cancer site**	Gastroenteric	70	46,4
	Gynecological	27	17,9
	Breast	20	13,2
	Lung	14	9,3
	Head and neck	1	0,7
	Rare	6	3,9
	Uro-genital	13	8,6
**Cancer stage**	1	3	2,0
	2	12	7,9
	3	24	15,9
	4	112	74,2
**Toxicity of treatments°**	0	72	47,7
	1	25	16,6
	2	38	25,2
	3	14	9,3
	4	2	1,3
**Intent of treatment**	Adjuvant	34	22,5
	NA*	3	2,0
	New adjuvant	3	2,0
	Palliative	111	73,5
**Response to treatment**	Absence of cancer	23	15,2
	Ongoing	58	38,4
	In progress	19	12,6
	Partial	22	14,6
	Stable	29	19,2
**Adherence after 3 months of treatment**	No	15	10,0
	Yes	136	90,0

**Legend: **The toxicity of treatment was scored from 0 (mild) to 4 (death), according to common toxicities criteria (CTC), version 4.0 [38]; available from: https://ctep.cancer.gov/protocoldevelopment/electronic_applications/ctc.htm#ctc_50.; NA* = not available data.

**Table 3:** Attributable burden on worsening QoL in patients with solid cancers: comparison with other chronic diseases.

**DISEASE**	**SF-12** **(mean±sd)**	ATTRIBUTABLE BURDEN ON QOL*	**COMPARISON WITH SOLID CANCER** **(one-way ANOVA)**
Major Depression [42]	33.8±9.2	5.6±3.6 (N=37)	F=0.6753, df 1,186,187 p=0.4123
Multiple Sclerosis [26]	29.5±7.3	7.0±3.5 (N=201)	F= 18.0766, df 1,350,351 p=0.000
Wilson’ Disease [41]	33.8±9.0	4.4±1.7 (N=23)	F= 0.0375, df 1,172,173 p= 0.8467
Carotidal atherosclerosis [40]	30.6±8.1	6.2±5.0 (N=46)	F= 2.0799, df 1,195,196 p= 0.1509
Celiac Disease [27]	35.83±5.72	2.4±1.0 (N=60)	F= 6.9304, df 1,209,210 p= 0.0091
Fibromyalgia [28]	26.43±6.04	11.02±5.56 (N=71)	F= 48.8075, df 1,220,221 p= 0.0000
Obsessive Compulsive Disorder [43]	35.4±6.9	2.9±6.0 (N=88)	F= 1.619, df 1,175,176 p= 0.205
PTSD [44]	36.3±6.1	3.9±1.0 (N=26)	F= 0.347, df 1,175,176 p= 0.557
Specific Phobia [45]	38.3±5.2	0.4±4.9 (N=28)	F=10.497 df 1,77,178 P=0.001
Solid cancer	32.34±6.764	4.67±6.64 (N=151)	-

**Legend**

*difference between the mean SF-12 score obtained in the groups of patients with several diseases in comparison with QoL scores obtained from the healthy general population.

**Table 4:** Association between worse QoL and moderate-severe depressive symptoms (PHQ-9≥14) controlling for cancer stage (1, 2, 3, 4).

-	**SF-12** **(mean±sd)**	**CANCER STAGE** **(mean±sd)**
**PHQ-9≥14 **	23.16±5.069	3.68±0.820
**PHQ-9<14 **	33.66±5.905	3.61±0.706
**One way ANOVA**	F=54.254; df 1,149,150 p= .000	F=0.59; df 1,149,150 p= .690

**Table 5:** Attributable burden on worsening QoL due to Major Depressive Disorder in people with solid cancers in comparison to that due to Major Depressive Disorder in other chronic diseases.

-	**Attributable Burden to Major Depressive Disorder**	**One-way ANOVA ** **F ** **(df)**	**p**
Solid Cancers (N=151)	10.1±5.7	Pivot	-
Multiple Sclerosis (N=201) [26]	2.9±7.4	98.85 (1,350,351)	<0.0001
Fibromyalgia (N=71) [28]	4.77±5.76	42.27 (1,220,221)	<0.0001
Wilson Disease (N=16) [41]	3.2±7.9	19.56 (1,165,166)	<0.0001
Celiac Disease (N=60) [27]	3.4±5.4	61.09 (1,201,210)	<0.0001
Carotidal Atherosclerosis (N=46) [40]	3.4±8.2	39.07 (1,195,196)	<0.0001

## Data Availability

The data supporting the findings of the article is available from the corresponding author [C.I.A.G], upon reasonable request.
